# Diabetes is a risk factor for high-dose methotrexate-associated AKI in lymphoma patients

**DOI:** 10.1080/0886022X.2020.1838926

**Published:** 2020-11-09

**Authors:** Yujia Wang, Li Wei, Yi Guan, Qian Wang, Qionghong Xie, Chuanming Hao

**Affiliations:** a Division of Nephrology, Huashan Hospital, and Nephrology Research Institute, Fudan University, Shanghai, China; b Division of Hematology, Huashan Hospital, Fudan University, Shanghai, China

**Keywords:** Acute kidney injury, diabetes, high-dose methotrexate, toxicity

## Abstract

**Purpose:**

The aim of the study was to investigate the incidence of acute kidney injury (AKI) occurring after high-dose methotrexate (HDMTX) administration and the role of type 2 diabetes (T2D) playing in the occurrence of AKI.

**Methods:**

We assessed associations between T2D along with other confounding factors mainly including baseline estimated glomerular filtration rate (eGFR), methotrexate (MTX) elimination and urine pH, and AKI occurrence. Patients who were diagnosed as primary central nervous system lymphoma with treatment of HDMTX and with eGFR ≥60 mL/min/1.73 m^2^ were enrolled in this study.

**Results:**

Of the 507 courses enrolled in this study, 132 courses have T2D. Lower baseline eGFR, delayed MTX elimination, lower urine pH, and higher incidence of AKI were observed in T2D group. Using univariate logistic regression, several confounding factors including baseline eGFR, hypertension, MTX elimination, and urine alkalinization statistically and clinically important were screened out. After adjusting for these factors, T2D remained an independent association with AKI occurrence. AKI outcome had no significant relationship with severe hematological toxicity or hepatotoxicity. AKI was associated with faster eGFR decline after a series of HDMTX treatment courses.

**Conclusions:**

Patients with T2D have a higher sensitivity to AKI when administrated with HDMTX. This conclusion addresses safety concerns for making chemotherapy regimen for this population.

## Introduction

Methotrexate (MTX), an antimetabolite disturbing the metabolism of folic acid, is widely used as a component of therapy for nononcologic diseases including psoriasis and rheumatoid arthritis and many types of cancers invading either solid organs or the hematological system [1–4]. Methotrexate can be administered in a wide range of doses ranging from 20 mg/m^2^ orally to 33,000 mg/m^2^ intravenously for different indications [5]. High-dose methotrexate (HDMTX), defined as >500 mg/m^2^ intravenous MTX administration, is important in the treatment of acute lymphoblastic leukemia, osteosarcoma, and lymphomas especially in the central nervous system [6]. With high effectiveness brought by HDMTX than low dose of MTX, significant toxicities including nephrotoxicity, hepatotoxicity, mucositis, bone marrow suppression, and neurotoxicity follow at the same time [6]. MTX is cleared mainly by kidneys through both glomerular filtration and tubular secretion [7,8]. Nephrotoxicity caused by HDMTX arises from precipitation of MTX leading to crystal nephropathy [9] and direct toxic effect of MTX to renal tubules [10]. MTX crystals are poorly soluble in urine with acidic pH, exposing the whole body in a high concentration of MTX and aggravating toxicities [6,9]. Acute kidney injury (AKI) occurred in 18.5–40% of patients receiving HDMTX treatment [11] and mortality was about 4.4% in patients developing AKI by HDMTX [12].

Type 2 diabetes (T2D) has become a common cause of chronic kidney disease (CKD) and end-stage renal disease (ESRD). An estimated 8.8% of the global population have diabetes and the number will increase to 10.4% by 2040 [13]. Among patients who were diagnosed as T2D, 40% of them developed diabetic kidney disease (DKD) [14]. Diabetes mellitus has been considered as an risk factor of AKI in patients who underwent open heartsurgery [15,16] and received iodinated contrast media [17]. Mechanisms including mitochondrial dysfunction, oxidative stress and inhibition of autophagy are considered to relate T2D to AKI [18–20]. However, little research has focused on the association of T2D and AKI in patients who receive HDMTX treatment. We performed this study in order to investigate the incidence of AKI occurring after HDMTX administration and the role of T2D playing in the occurrence of AKI.

## Methods

### Patients

A retrospective study was performed over a three year period from December 2016 to December 2019 in Division of Hematology in Huashan Hospital, Fudan University. The study was approved by the ethics committee of Huashan Hospital, Fudan University (KY2020-061). Adult patients (≥18 years old) with primary central nervous system lymphoma (PCNSL) who were treated with HDMTX were enrolled. Both newly diagnosed and relapse/refractory patients were eligible. Diagnoses of PCNSL were determined by surgical resection and biopsy performed in our hospital. HDMTX was defined as >500 mg/m^2^ intravenous MTX administration. Patients without complete information and who had a history of CKD 3–5 stage and estimated glomerular filtration rate (eGFR) < 60 mL/min/1.73 m^2^ before the initiation of therapy were excluded. EGFR was calculated based on 2009 CKD-EPI equation.

### Clinical data

Data for each course of HDMTX administration were collected as an individual case through a retrospective electronic chart review and a single patient received HDMTX treatment once or more. The demographic characteristics (age and sex) and diabetes mellitus (DM) and hypertension history were recorded. Height and weight were measured according to a standard protocol, and body mass index (BMI) was calculated. The following baseline laboratory parameters before HDMTX administration were recorded: hemoglobin, white blood cell (WBC), neutrophil percentage (N%), platelet count (PLT), alanine aminotransferase (ALT), aspartate aminotransferase (AST), total bilirubin, albumin, and serum creatinine (SCr). The laboratory parameters were examined using an automatic chemical analyzer. Treatment regimens including MTX dose and other concommitant chemotherapy drugs use were collected. MTX was administered intravenously. All patients received adequate hydration, urine alkalinization, and leucovorin. MTX plasma concentration at 24, 48, and 72 h after injection and simultaneous urine pH were collected. Concomitant medications including antibiotics, proton pump inhibitor (PPI), angiotensin-converting enzyme/angiotensin receptor blocker (ACEI/ARB), and mannitol were also collected.

Diagnosis of T2D was based on medical history, laboratory tests such as fasting blood glucose and HbA1C, and the drugs that were used to treat T2D. Diagnosis of hypertension was based on medical history and antihypertensive drugs. SCr within 3 days before each treatment was recorded as a baseline. The maximum SCr elevation was calculated as from baseline to the peak value within 72 h after MTX administration. AKI was defined according to KDIGO criteria with an increase of SCr by 26.5 mmol/L within 48 h as well as an increase to 1.5 times baseline within 7 days as threshold for diagnosis [21]. 72-h AKI recovery was defined as SCr falling to baseline SCr within 72 h (a fluctuation within 20% allowed). Hematological toxicity and hepatotoxicity were assessed using CTCAE version 4.0.

### Statistical analysis

The continuous variables were expressed as means and standard deviation (SD) or median and quartile. They were analyzed with Student *t* test if in normal distribution, while with Mann–Whitney tests if in abnormal distribution. The categorical variables were expressed as numbers and percentages and analyzed with chi-squared tests. Serum creatinine at all time points during one treatment courses was recorded. The variation trend of SCr of diabetic and non-diabetic ones and the decline trend of eGFR of AKI and non-AKI ones were respectively compared by two-way repeated measures ANOVA. Univariate logistic regression was used to analyze the risk factors of AKI. Multivariable logistic regression was further used to determine independent risk factors of AKI. Statistical analyses were performed with SPSS v 25.0. *p* values as 0.05 was considered to be significant.

## Results

### Patients’ characteristics

**Figure 1.**
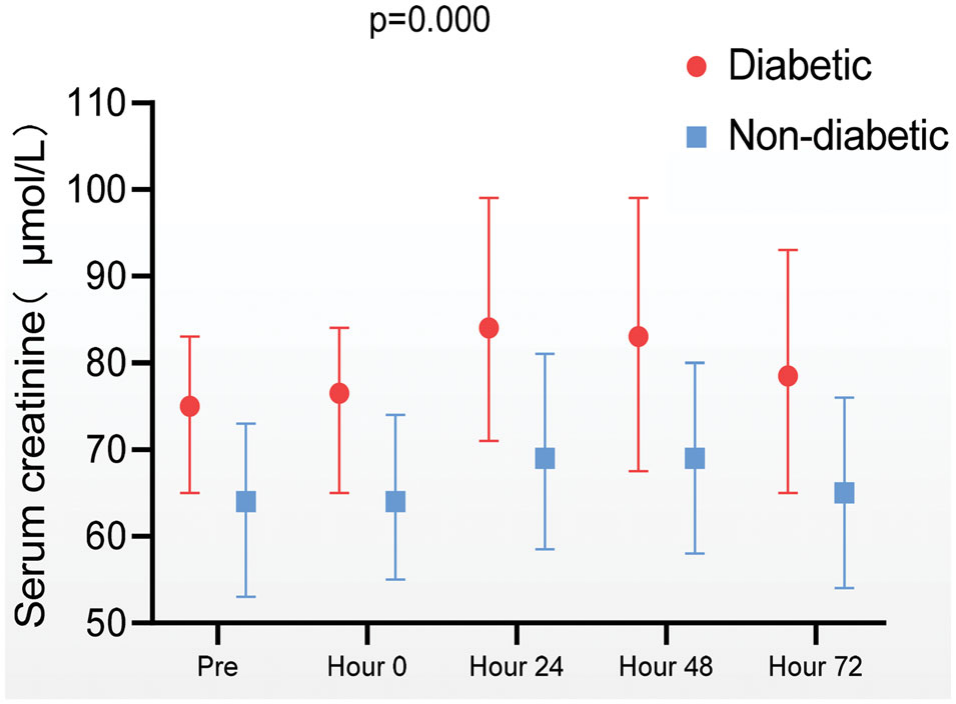
Serum creatinine elevation during the course of the diabetic and nondiabetic group.

A total of 117 patients aged between 27 and 82 years old with 64.96% of male were enrolled in this study (Table 1). Twenty-five patients had T2D. Compared to non-T2D patients, patients with T2D had a higher prevalence of hypertension. There were no significant differences in baseline eGFR or proteinuria before the first treatment between the T2D and non-T2D patients. The number of treatment courses received by a single patient ranged from 1 to 10 times. Fifty patients (42.73%) of all received 1–3 courses of HDMTX treatment, 46 patients (39.32%) received 4–6 courses and 21patients (17.95%) received more than six courses. Among the 117 patients, 65 developed AKI during HDMTX treatment at least once. Diabetic patients had more AKI episodes than nondiabetic ones. Diabetic patients showed higher SCr at any time point during MTX treatment course (Figure 1).

**Table 1.** Baseline characteristics of patients.

	Total (*n* = 117)	T2D (*n* = 25)	Non-T2D (*n* = 92)	*p* Value
Age (years)	60.0 [51.0–65.0]	61.0 [54.0–67.5]	59.0 [51.0–65.0]	0.317
Male (*n*, %)	76 (64.96)	20 (80.00)	56 (60.86)	0.075
BMI (kg/m^2^)	23.41 ± 2.74	23.98 ± 3.262	23.25 ± 3.98	0.259
BSA (m^2^)	1.83 [1.71–1.91]	1.87 [1.82–1.91]	1.80 [1.70–1.91]	0.086
Proteinuria (*n*, %)	0 (0.00)	0 (0.00)	0 (0.00)	–
Baseline eGFR (ml/min/1.73m^2^)	101.99 [93.78–108.02]	97.52 [89.56–107.39]	102.60 [96.23–108.59]	0.089
Hypertension (*n*, %)	26 (22.22)	12 (48.00)	14 (15.21)	0.000
Number of courses (*n*, %)				0.074
1–3	50 (42.73)	11 (44.00)	39 (42.39)	
4–6	46 (39.32)	6 (24.00)	40 (43.48)	
>6	21 (17.95)	8 (32.00)	13 (14.13)	
Number of AKI episodes (*n*, %)				
0	52 (44.44)	5 (20.00)	47 (51.09)	0.000
1	38 (32.48)	5 (20.00)	33 (35.87)	
>1	27 (23.08)	15 (60.00)	12 (13.04)	

AKI: acute kidney injury; BMI: body mass index; BSA: body surface area; eGFR: estimated glomerular filtration rate; T2D: type 2 diabetes.

A total of 507 HDMTX treatment courses were recorded and among them 132 treatment courses occurred in patients with T2D (Table 2). Compared to non-T2D group, T2D group had lower baseline eGFR, higher plasma MTX concentration, and lower urine pH. AKI was much more often and more intensive following each treatment course in T2D group than in non-T2D group. The rate of 72-h recovery of AKI was significantly lower in T2D patients, despite a lower median dose of MTX administration. The use of medicines that could interfere with MTX clearance was comparable between the diabetic and non-diabetic patients.

**Table 2.** Characteristics before treatment, MTX and AKI information of courses.

Characteristics	Total (*n* = 507)	T2D (*n* = 132)	Non-T2D (*n* = 375)	*p* Value
eGFR (ml/min/1.73m^2^)	98.86 [90.37–106.78]	91.96 [82.72–103.35]	100.53 [93.18–108.44]	0.000
Hemoglobin (g/L)	125.65 ± 14.82	128.64 ± 11.48	124.60 ± 15.71	0.002
WBC (×10^9^/L)	6.14 [4.79–7.95]	6.37 [5.03–8.09]	6.02 [4.66–7.84]	0.230
N%	61.2 [53.1–68.4]	63.2 [56.2–69.2]	60.5 [52.4–68.0]	0.044
PLT (×10^9^/L)	203.0 [165.0–248.0]	184.5 [151.2–217.7]	209.0 [169.0–255.0]	0.000
ALT (U/L)	20.0 [13.0–32.0]	21.0 [15.0–33.0]	20.0 [12.0–32.0]	0.230
AST (U/L)	19.0 [15.0–24.0]	18.0 [15.0–23.0]	19.0 [15.0–25.0]	0.145
Total bilirubin (μmol/L)	7.95 [6.20–10.20]	8.50 [7.00–10.47]	7.65 [6.00–10.10]	0.013
Albumin (g/L)	40.0 [37.0–43.0]	40.5 [38.0–43.0]	40.0 [37.0–43.0]	0.157
Treatment regimen (*n*, %)				<0.001
MTX + DXM	38 (7.49）	9 (6.82)	29 (7.73)	
MTX + DXM + Rutiximab	252 (49.70)	87 (65.91)	165 (44.00)	
MTX + DXM + Idarubicin	14 (2.76)	1 (0.76)	13 (3.47)	
MTX + DXM + Doxorubicin	41 (8.09)	4 (3.03)	37 (9.87)	
MTX + DXM + Rutiximab, Idarubicin	25 (4.93)	7 (5.30)	18 (4.80)	
MTX + DXM + Cydarabine	24 (4.73)	0 (0)	24 (6.40)	
MTX + DXM + Vindesine, Cyclophosphamide, Doxorubicin liposome	20 (3.95)	0 (0)	20 (5.33)	
MTX + DXM + Vindesine, Cyclophosphamide, Epirubicin	29 (5.72)	8 (6.06)	21 (5.60)	
Others	64 (12.63)	16 (12.12)	48 (12.80)	
MTX dose (g/m^2^)	4.13 [3.33–7.70]	3.51 [3.24–7.89]	4.22 [3.37–7.40]	0.972
MTX Elimination				
MTX concentration at 24 h (μmol/L)	1.78 [1.10–3.42]	2.00 [1.53–4.26]	1.59 [1.01–3.25]	0.000
MTX concentration at 48 h (μmol/L)	0.26 [0.19–0.39]	0.33 [0.24–0.48]	0.24 [0.18–0.35]	0.000
MTX concentration at 72 h (μmol/L)	0.14 [0.08–0.19]	0.16 [0.10–0.22]	0.12 [0.06–0.17]	0.000
Urine pH ≥7 at both 0 and 24 h (n, %)	359 (70.80)	79 (59.84)	280 (74.67)	0.001
Concomitant drug uses (*n*, %)				
Antibiotics				0.526
Cephalosporin	27 (5.33)	7 (5.30)	20 (5.33)	
Penicillins	3 (0.59)	0 (0)	3 (0.80)	
Quinolones	25 (4.93)	3 (2.27)	22 (5.87)	
Imipenem/meropenem	7 (1.38)	1 (0.76)	6 (1.60)	
A combination of two kinds of antibiotics	15 (2.95)	5 (3.79)	10 (2.67)	
Non-antibiotics				
Mannitol	66 (13.01)	13 (9.84)	53 (14.13)	0.208
ACEI/ARB	34 (6.71)	13 (9.84)	21 (5.60)	0.093
PPI	465 (91.72)	118 (89.39)	347 (92.53)	0.260
AKI (*n*, %)	99 (19.52)	43 (32.57)	56 (14.93)	0.000
Stage 1	82 (82.82)	33 (76.74)	49 (87.50)	
Stage 2	14 (14.14)	9 (20.93)	5 (8.93)	
Stage 3	3 (3.04)	1 (2.33)	2 (3.57)	
SCr elevation (% of baseline SCr)	21.41 [11.68–37.77]	28.20 [11.88–43.84]	20.51 [11.36–35.00]	0.033
72-h recovery (*n*, %)	347 (68.44)	80 (60.60)	267 (71.20)	0.024

ACEI/ARB: angiotensin-converting enzyme/angiotensin receptor blocker; AKI: acute kidney injury; ALT: alanine aminotransferase; AST: aspartate aminotransferase; eGFR: estimated glomerular filtration rate; MTX: methotrexate; PLT: platelet; PPI: proton pump inhibitor; SCr: serum creatinine; T2D: type 2 diabetes; WBC: white blood cell count.

### T2D was an independent risk factor of AKI

To examine the risk factors of AKI, all the 507 HDMTX treatment courses were divided into two groups (Table 3). Compared to the non-AKI group, AKI group had more T2D, hypertension, lower baseline eGFR, higher plasma MTX concentrations, lower urine pH and more concomitant use of AECI/ARB during the courses.

**Table 3.** Risk factors for AKI.

	AKI (*n* = 99)	non-AKI (*n* = 408)	OR	*p* Value
Age (years)	61.00 [54.00–65.00]	60.00 [50.25–65.00]	1.013 [0.992–1.034]	0.215
Male (*n*, %)	68 (68.68)	271 (66.42)	0.902 [0.563–1.445]	0.668
BMI (kg/m^2^)	23.88 [22.06–25.48]	23.81 [21.39–24.80]	0.996 [0.970–1.024]	0.796
BSA (m^2^)	1.87 [1.77–1.92]	1.81 [1.69–1.91]	2.354 [0.781–7.093]	0.128
eGFR (ml/min/1.73m^2^)	97.33 [87.21–104.27]	99.07 [91.54–107.43]	0.984 [0.969–1.000]	0.046
Hemoglobin (g/L)	125.49 ± 14.85	125.69 ± 14.83	0.999 [0.984–1.014]	0.906
WBC (×10^9^/L)	5.66 [4.62–8.07]	6.21 [4.84–7.93]	0.956 [0.882–1.036]	0.276
N%	61.40 [53.10–67.20]	61.10 [53.15–68.60]	0.994 [0.978–1.011]	0.521
PLT (×10^9^/L)	188.00 [167.00–239.00]	203.50 [163.25–249.00]	0.999 [0.996–1.002]	0.480
ALT (U/L)	22.00 [14.00–36.00]	20.00 [13.00–32.00]	1.002 [0.991–1.012]	0.768
AST (U/L)	18.00 [15.00–26.00]	19.00 [15.00–24.00]	1.001 [0.978–1.023]	0.951
Total bilirubin (μmol/L)	8.00 [6.60–10.00]	7.90 [6.10–10.30]	1.016 [0.971–1.064]	0.491
Albumin (g/L)	40.00 [37.00–44.00]	40.00 [37.00–43.00]	1.010 [0.959–1.064]	0.699
Diabetes (*n*, %)	43 (43.43)	89 (21.81)	2.752 [1.735–4.367]	0.000
Hypertension (*n*, %)	31 (31.31)	87 (21.32)	1.682 [1.034–2.736]	0.036
Treatment regimen (*n*, %)				0.412
MTX + DXM	10 (10.10)	28 (6.86)	1.548 [0.595-4.029]	
MTX + DXM + Rutiximab	54 (54.55)	198 (48.53)	1.182 [0.589-2.371]	
MTX + DXM + Idarubicin	3 (3.03)	11 (2.70)	1.182 [0.285-4.902]	
MTX + DXM + Doxorubicin	4 (4.04)	37 (9.07)	0.469 [0.140-1.567]	
MTX + DXM + Rutiximab, Idarubicin	6 (6.06)	19 (4.66)	1.368 [0.450-4.160]	
MTX + DXM + Cydarabine	3 (3.03)	21 (5.14)	0.619 [0.158-2.419]	
MTX + DXM + Vindesine, Cyclophosphamide, Doxorubicin liposome	1 (1.01)	19 (4.66)	0.228 [0.028-1.875]	
MTX + DXM + Vindesine, Cyclophosphamide, Epirubicin	6 (6.06)	23 (5.64)	1.130 [0.378-3.383]	
Others	12 (12.12)	52 (12.74)	Ref	
MTX dose (g/m^2^)	4.73 [3.39–7.82]	3.90 [3.32–7.57]	1.076 [0.972–1.191]	0.157
MTX concentration >1 μmol/L at 48 h and >0.1 μmol/L at72 h (*n*, %)	28 (28.28)	10 (2.45)	15.696 [7.304–33.727]	0.000
Urine pH ≥7 at both 0 and 24 h (*n*, %)	61 (61.62)	298 (73.04)	0.593 [0.374–0.939]	0.025
Concomitant drug uses (*n*, %)				
Mannitol	14 (14.14)	52 (12.75)	1.128 [0.597–2.130]	0.711
ACEI/ARB	12 (12.12)	22 (5.39)	2.420 [1.154–5.077]	0.019
PPI	95 (95.95)	370 (90.69)	2.439 [0.850–7.003]	0.098

ACEI/ARB: angiotensin-converting enzyme/angiotensin receptor blocker; AKI: acute kidney injury; ALT: alanine aminotransferase; AST: aspartate aminotransferase; BMI: body mass index; BSA: body surface area; eGFR: estimated glomerular filtration rate; MTX: methotrexate; OR: odds ratio; PLT: platelet; PPI: proton pump inhibitor; WBC: white blood cell count.

In univariate analysis, T2D was a risk factor for AKI. The effect of T2D on AKI was further analyzed after adjusting for a series of potential confounding factors (Table 4). Model 1 was adjusted for age and gender, Model 2 for age, gender, hypertension, and baseline eGFR, and Model 3 further for MTX concentration and urine pH value. In all adjusted models, T2D remained independently associated with the incidence of AKI. The odds ratio was 2.998 [1.692–5.310] (*p* = 0.000) in the most fully adjusted Model 3.

**Table 4.** T2D associates with AKI across stratified analyses.

	MODEL 1 (adjusted for demographics)	MODEL 2 (adjusted for demographics, hypertension and eGFR)	MODEL 3 (adjusted for demographics, hypertension, eGFR, MTX elimination and urine alkalinization)
T2D	2.747 [1.682–4.487] (0.000)	2.509 [1.489–4.227] (0.001)	2.998 [1.692–5.310] (0.000)
Age	1.004 [0.982–1.027] (0.714)	0.996 [0.968–1.026] (0.800)	0.999 [0.968–1.031] (0.950)
Gender	1.080 [0.645–1.810] (0.770)	1.096 [0.646–1.861] (0.733)	1.169 [0.656–2.081] (0.597)
eGFR before treatment	–	0.992 [0.971–1.012] (0.424)	1.002 [0.980–1.025] (0.851)
Hypertension		1.217 [0.716–2.068] (0.469)	1.021 [0.565–1.844] (0.946)
MTX elimination	–	–	16.086 [7.172–36.078] (0.000)
urine alkalinization	–	–	0.813 [0.480–1.380] (0.444)

AKI: acute kidney injury; eGFR: estimated glomerular filtration rate; MTX: methotrexate; T2D: type 2 diabetes.

### AKI occurrence was not associated with other toxicities

Toxicities of other systems after HDMTX treatment were recorded in Table 5. The most common grades 3–4 toxicities were hepatotoxicity. Seventy-two courses of 507 (14.20%) experienced grades 3–4 elevated AST or AST or TB. Ten courses (1.97%) experienced grades 3–4 toxicity of hematological system, with four having anemia, three having neutropenia, and three having thrombocytopenia. Twenty-six courses (5.13%) developed infections after the treatment course. No difference in these toxicities between AKI and non-AKI group was observed.

**Table 5.** The occurrence of grades 3–4 hematological toxicity and infections after HDMTX treatment, hepatotoxicity.

	Total (*n* = 507)	AKI (*n* = 99)	non-AKI (*n* = 408)	*p* Value
Anemia (*n*, %)	4 (0.79)	0 (0)	4 (0.98)	0.418
Neutropenia (*n*, %)	3 (0.59)	2 (2.02)	1 (0.25)	0.099
Thrombocytopenia (*n*, %)	3 (0.59)	2 (2.02)	1 (0.25)	0.099
Elevated ALT/AST/TB (*n*, %)	72 (14.20)	14 (14.14)	58 (14.22)	0.985
Infections (*n*, %)	26 (5.13)	9 (9.09)	17 (4.17)	0.070

### Effects of AKI on eGFR decline after several courses of HDMTX treatment

**Figure 2.**
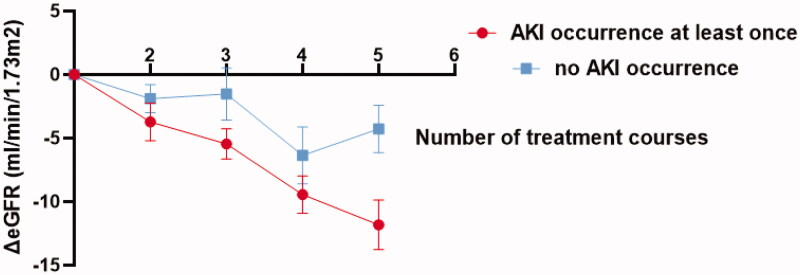
Effects of AKI on eGFR decline during 5-courses HDMTX treatment.

Fifty-five patients completed at least five HDMTX treatment courses. Thirty-six of them developed AKI at least once during all courses. A downward trend of eGFR during the first five courses was observed (*F* = 9.885 *p* < 0.001). The number of treatment courses and AKI occurrence had no interaction on eGFR change (*F* = 1.837 *p* = 0.136). Patients who occurred AKI at least once had more intensive eGFR decline than those who did not occur AKI at all during the first five courses (*F* = 4.070 *p* = 0.049) (Figure 2).

## Discussion

High dose of MTX is associated with AKI. In this retrospective study of HDMTX treatment in PCNSL patients, we found a positive correlation between T2D and incidence of AKI, and T2D is an independent risk factor for HDMTX-associated AKI in PCNSL patients after adjusting other risk factors as baseline eGFR, MTX elimination, and urine pH [9,22].

The increasing prevalence of DKD has been seen recently, paralleling the dramatic rise of the diabetic population worldwide [23]. It is widely accepted that DKD predisposes patients with AKI and retards the recovery [18]. Albuminuria and declining eGFR are two major manifestations in DKD, which are recommended to be measured in order to screen and evaluate the progression of kidney function in patients with T2D [24]. Robust associations between elevated albuminuria and decreased eGFR were observed in previous studies [25,26]. In patients enrolled in our study, proteinuria was rarely recorded even in those with T2D. In the early stage of diabetes when DKD cannot be diagnosed clinically, dysfunctional renal hemodynamics as a result of metabolite disorders may predispose and sensitize diabetic patients to AKI [23]. It has been shown by other cohorts that AKI was substantially more likely to happen in patients with eGFR < 60 mL/min per 1.73 m^2^ [25–27]. In our study, AKI incidence was still as high as 25.34% even in patients with eGFR ≥ 60 mL/min per 1.73 who treated with HDMTX. In T2D patients, AKI incidence was 32.57%, much higher than that in non-T2D patients (14.93%). Additionally, SCr elevation was more intensive and AKI recovery rate within 72 h was lower in T2D patients, comparing with non-T2D patients. AKI is closely associated with the increased risk of developing CKD [28]. The incompletely recovered tubules exhibit persistent, unregulated, and increasing profibrotic signaling along multiple pathways [29]. We observed that patients who had AKI at least once showed faster eGFR decline after a series of HDMTX treatment courses, which indicated these patients were at higher risk of developing CKD further.

Consistent with a large number of previous studies, MTX elimination and urine acidity were also risk factors of renal toxicities induced by HDMTX [5,6]. Therefore, daily monitoring of MTX concentration and sufficient use of sodium bicarbonate to achieve alkaline urine during HDMTX treatment is needed in clinical practice. In diabetic patients, we found serum MTX concentrations were significantly higher and urine pH value was lower at 0 and 24 h after MTX administration despite lower MTX dose and aggressive urine alkalinization. Lower urine pH value was observed in diabetic patients in other studies focused on nephrolithiasis, which showed that T2D patients had higher net acid excretion (NAE) and lower proportion of NAE as NH4+ measured in urine, suggesting impaired renal ammonium production and excretion in diabetes [30–32]. The mechanism remains uncertain although some animal and cell models tried to explain it with lipid accumulation in renal tubular cells as a result of T2D [33,34]. Acidic urine decreases the solubility of MTX and its metabolites and promotes the formation of crystals within renal tubules, which causes delayed MTX elimination [6]. In our study, multivariate regression analysis showed diabetes was an independent risk factor of AKI, while urine pH value not, suggesting lower urine pH value was possibly a result of diabetes.

There are several limitations to our study. First, this was a retrospective study and AKI was diagnosed through reviewing SCr elevation in electronic charts, which may mistake AKI causes to HDMTX in some cases. Second, medications associated with MTX excretion, such as nonsteroidal anti-inflammatory drugs (NSAIDs), were not analyzed. Third, the treatment responses were not assessed in the study. Finally, we presented data on the associations between diabetes along with other characteristics and AKI using odds ratios, which may overestimate the relative risk when the event of interest is common.

## Conclusion

We found T2D is an independent risk factor of AKI in patients administered with HDMTX and it almost doubles the risk of AKI occurrence. Before the presence of proteinuria and decline of eGFR, T2D substantially predisposes patients to AKI, indicating potential kidney injury even before the clinical diagnosis of DKD. T2D patients had lower urine pH value which contributed to the delayed MTX elimination and therefore AKI occurrence. In addition, patients developing AKI at once had a poorer renal function after five treatment courses. This study addresses safety concerns to clinical doctors who make regimens for PCNSL patients with T2D and provide therapeutic benefits in this patient population. More aggressive supportive care and timely recognition of renal toxicities are significant for diabetic patients during HDMTX treatment courses.
